# Machine-learning approach on echocardiography to improve the detection of transthyretin amyloid cardiomyopathy: GRAAL algorithm

**DOI:** 10.1093/ehjdh/ztag022

**Published:** 2026-03-25

**Authors:** Antoine Fraix, Olivier Huttin, Claire Lacomblez, Nathalie Pace, Pierre-Yves Marie, Damien Mandry, Marine Claudin, Nicolas Sadoul, Laura Filippetti, Erwan Donal, Olivier Lairez, Emmanuelle Lointier, Amira Zaroui, Thibaud Damy, Christine Selton-Suty, Nicolas Girerd

**Affiliations:** Cardiology Department, Institut Lorrain du Cœur et des Vaisseaux, CHRU de Nancy, Site Brabois, 54500 Vandoeuvre, France; Cardiology Department, Institut Lorrain du Cœur et des Vaisseaux, CHRU de Nancy, Site Brabois, 54500 Vandoeuvre, France; INSERM, Centre D’Investigations Cliniques-1433, and INSERM U1116, Université de Lorraine, CHRU de Nancy, 54500 Vandoeuvre, France; F-CRIN INI-CRCT, (Cardiovascular and Renal Clinical Trialists), Université de Lorraine, Nancy, France; Cardiology Department, Institut Lorrain du Cœur et des Vaisseaux, CHRU de Nancy, Site Brabois, 54500 Vandoeuvre, France; Cardiology Department, Institut Lorrain du Cœur et des Vaisseaux, CHRU de Nancy, Site Brabois, 54500 Vandoeuvre, France; Cardiology Department, Institut Lorrain du Cœur et des Vaisseaux, CHRU de Nancy, Site Brabois, 54500 Vandoeuvre, France; Cardiology Department, Institut Lorrain du Cœur et des Vaisseaux, CHRU de Nancy, Site Brabois, 54500 Vandoeuvre, France; Cardiology Department, Institut Lorrain du Cœur et des Vaisseaux, CHRU de Nancy, Site Brabois, 54500 Vandoeuvre, France; Cardiology Department, Institut Lorrain du Cœur et des Vaisseaux, CHRU de Nancy, Site Brabois, 54500 Vandoeuvre, France; Cardiology Department, Université de Rennes-1, 35000 Rennes, France; Cardiology Department, Rangueil University Hospital, 31400 Toulouse, France; Cardiology Department, Rangueil University Hospital, 31400 Toulouse, France; French Referral Centre for Cardiac Amyloidosis, Amyloidosis Mondor Network, GRC Amyloid Research Institute, CHU Henri Mondor, Creteil 94000, France; French Referral Centre for Cardiac Amyloidosis, Amyloidosis Mondor Network, GRC Amyloid Research Institute, CHU Henri Mondor, Creteil 94000, France; Cardiology Department, Institut Lorrain du Cœur et des Vaisseaux, CHRU de Nancy, Site Brabois, 54500 Vandoeuvre, France; Cardiology Department, Institut Lorrain du Cœur et des Vaisseaux, CHRU de Nancy, Site Brabois, 54500 Vandoeuvre, France; INSERM, Centre D’Investigations Cliniques-1433, and INSERM U1116, Université de Lorraine, CHRU de Nancy, 54500 Vandoeuvre, France; F-CRIN INI-CRCT, (Cardiovascular and Renal Clinical Trialists), Université de Lorraine, Nancy, France

**Keywords:** Amyloidosis, Transthyretin amyloid cardiomyopathy, Machine learning, Echocardiography, Screening

## Abstract

**Aims:**

Transthyretin amyloid cardiomyopathy (ATTR-CM) is an increasingly recognized cause of heart failure, yet detection remains challenging due to its echocardiographic similarities with age- and hypertension-related cardiac remodelling.

**Methods and results:**

We retrospectively included 260 patients (76.5 ± 12.9 years old, 59.6% male) referred for suspected ATTR-CM. A supervised machine-learning diagnosis algorithm differentiating patients with (*n* = 111) and without (*n* = 149) ATTR-CM based on echocardiographic data, and subsequently validated in an external multicentre cohort of 454 patients (76.3 ± 12.6 years old, 69.1% male). Patients with ATTR-CM had a lower systolic function [left ventricular ejection fraction 47 ± 11 vs. 54 ± 12%, *P* < 0.00; global longitudinal strain (GLS) 11.0 ± 3.7 vs. 14.1 ± 4.5%, *P* < 0.001] and more significant relative apical longitudinal sparing (RALS) (1.5 ± 1.2 vs. 0.9 ± 0.4, *P* < 0.001) compared with controls. Machine learning identified right ventricular free wall thickness (RVFWT), RALS, GLS, and LV mass index as key variables for detecting ATTR-CM [AUC 0.90 (0.86–0.94); *P* < 0.001]. These variables enhanced diagnostic accuracy compared with the increased wall thickness guideline score [increase in C-index of 0.17 (0.11–0.23), *P* < 0.001]. Diagnostic performance was confirmed in the validation multicentre cohort [AUC of 0.83 (0.80–0.87), *P* < 0.001]

**Conclusion:**

The simple GRAAL algorithm (**G**LS, **R**VFWT, **A**pical sp**A**ring, **L**V Mass) enhances detection accuracy for ATTR-CM and improves patient selection for bone scintigraphy.

## Graphical Abstract

**Graphical Abstract:**
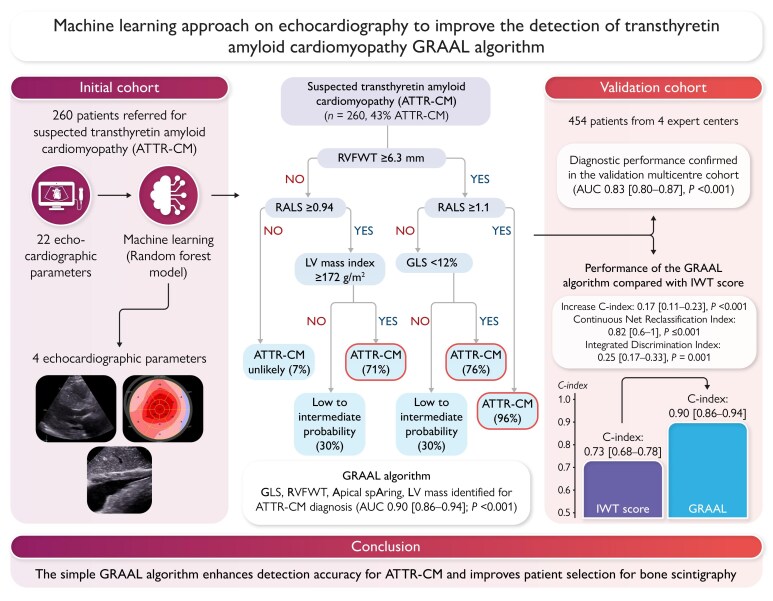

## Introduction

Transthyretin amyloid cardiomyopathy (ATTR-CM) is an increasingly recognized cause of heart failure (HF) particularly in the elderly. In recent studies, ATTR-CM represented 26% of patients aged 80 and over with hypertrophic cardiomyopathy phenotype as well as up to 8–12% of patients with HF with preserved ejection fraction (HFpEF) or aortic stenosis.^1,2^ This rising prevalence is related to the advent of bone scintigraphy as a non-invasive tool providing definitive diagnosis for ATTR-CM and increasing clinical awareness.^3,4^ Nevertheless, the most effective detection strategy for ATTT-CM remains an issue of debate.^5–7^

Comorbidities including hypertension, renal failure, and aortic stenosis can result in cardiac remodelling patterns that closely resemble ATTR-CM, making detection difficult in elderly populations.^7,8^ To enhance detection, myocardial deformation parameters and multimodal imaging-based algorithms—such as the RAISE score—have been proposed in both general and targeted populations.^2,5–7^ However, most of the echocardiographic ‘red flags’ such as left ventricular hypertrophy, diastolic dysfunction and ‘apical sparing’ have a low discriminative value in non-selected populations with a high comorbidity burden.^7–11^ A multiparametric approach hence appears essential to improve the diagnostic value of echocardiography.

Given the challenges of traditional statistical methods in identifying patients at high risk for ATTR-CM, machine-learning and artificial intelligence (AI) approach could be particularly useful. Huda *et al.* proposed a screening approach based on ICD codes using a random forest-based algorithm to screen patients most likely to have ATTR-CM.^12^ This approach allows for broad initial screening with echocardiographic assessment as a second step before confirmatory diagnostic testing, primarily bone scintigraphy.

The primary objective of this study was to develop and validate a machine-learning-based algorithm incorporating structural and functional echocardiographic parameters to improve ATTR-CM detection.

## Material and methods

### Initial cohort

A retrospective cohort study was conducted at the University Hospital of Nancy. Consecutive patients referred for suspected ATTR-CM between 2011 and 2021 were evaluated. From the initial population of 390 patients, 81 were excluded due to AL-CM and 5 to AA-CM, and a further group of 44 patients were excluded due to incomplete echocardiographic or diagnosis data. Patients underwent a complete evaluation including structural and functional transthoracic echocardiography (TTE), laboratory measurements and bisphosphonate scintigraphy. ATTR-CM diagnosis was based on the Gilmore *et al*. non-invasive diagnostic approach using the combination of TTE features indicative of ATTR-CM, with Perugini’s grade 2 or 3 cardiac uptake on scintigraphy with 99mtechnetium (99mTc)-labelled radiotracers [99mTc-hydroxymethylene diphosphonate, 99mTc-3,3-diphosphono-1,2-propanodicarboxylic acid (DPD) and 99mTc-pyrophosphate] in the absence of monoclonal gammopathy.^3,4^ Three patients, prior to 2016, did not undergo bone scintigraphy but benefited from histological and magnetic resonance imaging (MRI) confirmation. The 8 ATTR-CM variants were distributed as follows: 3 V30M, 3 V122I, 1 P64I, and 1 A36P. A total of 119 patients underwent a biopsy with a large majority of accessory salivary gland (*n* = 104, 87%) and a minority of endomyocardial biopsy (*n* = 10, 8%). Biopsies (one or several) confirm ATTR deposits in 61 (17%) of cases. This study complied with the Declaration of Helsinki and was approved by local regulatory committees.

### Validation cohort

The validation cohort included 454 patients from four University hospitals: Nancy (*n* = 59), Toulouse (*n* = 75), Rennes (*n* = 113), and Creteil (*n* = 207) who undergoing a diagnostic workup for ATTR-CM. ATTR-CM diagnosis was confirmed using the same non-invasive approach.^4^

### Echocardiography

**Figure 1:**
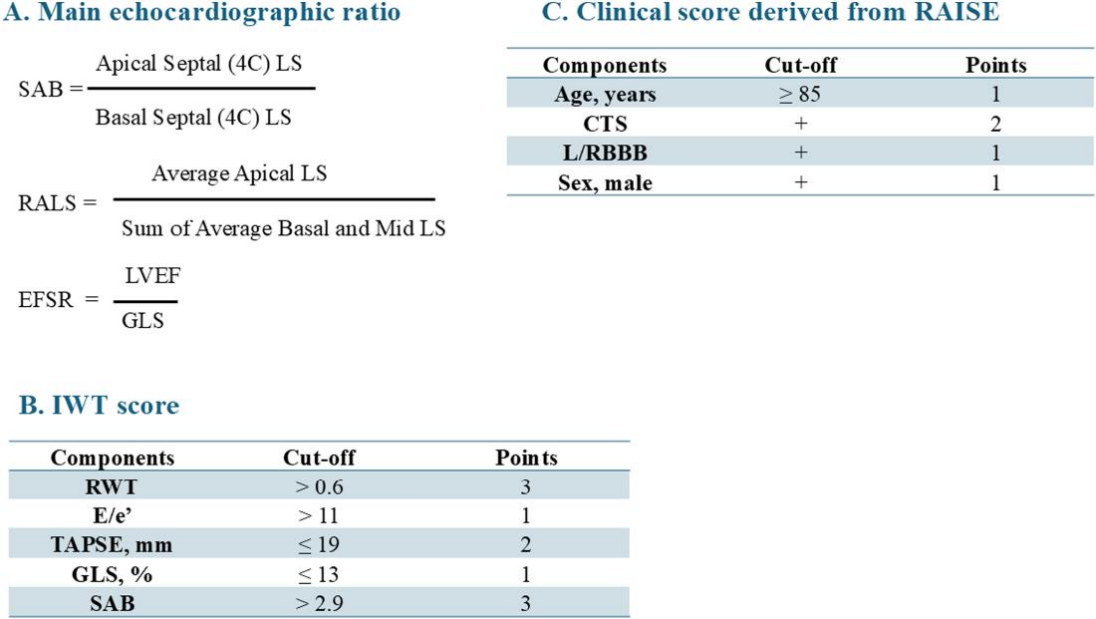
Main echocardiographic ratio and score for ATTR-CM diagnosis. ATTR-CM: transthyretin amyloid cardiomyopathy, CTS: carpal tunnel syndrome, EFSR: ejection fraction strain ratio, (G)LS: (global) longitudinal strain, LBBB: left bundle branch block, RALS: relative apical longitudinal sparing, RBBB: right bundle branch block, RWT: relative wall thickness, SAB: septal apical to base ratio.

Echocardiographic data were acquired using standardized imaging protocols at the University Hospital of Nancy by experienced cardiologists using commercially available standard ultrasound scanners (Vivid 7, 9 or E95, GE Medical Systems, Milwaukee, WI, USA) with a 2.5-MHz transducer, and were all reviewed offline using dedicated software (Echo PAC® PC version 110.1.0, GE Healthcare) according to current recommendations.^13–15^ Details regarding measurements are summarized in Supplementary material online, *Appendix S1*. Right ventricular free wall thickness (RVFWT) was measured in the subcostal or left parasternal view at end-diastole, in the middle segment, using two-dimensional (2D) imaging with an increase defined as wall thickness > 5 mm.^15^ Different specifics ratio has been calculated including: the septal apical to base ratio (SAB), the ejection fraction strain ratio (EFSR), and relative apical longitudinal sparing (RALS) and resumed in *Figure 1*.^5,16^ The increased wall thickness (IWT) score was adapted from the study by Boldrini *et al.*, using a 10-point scale with a diagnostic threshold set at ≥8 and resumed in *Figure 1*.^6^ Acquisition of echocardiographic data was carried out at the time of the diagnostic suspicion. The review and the realization of complementary measures were carried out, blind to the results of these additional tests.

### Radionuclide bone scintigraphy with technetium-labelled bisphosphonates (TcDPD)

Patients were scanned 3 h after intravenous injection of 500 to 800 MBq 99mTc-DPD using a gamma camera equipped with low-energy high-resolution collimators and conventional methodology. Cardiac retention was defined by experts in each centre according to the visual grading scale on planar imaging defined by Perugini *et al*. using the following grading system: grade 0 = absent cardiac uptake; grade 1 = mild uptake less than bone; grade 2 = moderate uptake equal to bone; and grade 3 = high uptake greater than bone.^3^

### Machine-learning approach: decision tree construction

We employed a supervised decision tree-based machine-learning algorithm to predict the diagnosis of ATTR-CM. The algorithm was implemented using the Rpart package in R, which is designed for recursive partitioning and regression trees. ATTR-CM diagnosis was used as class and 22 structural and functional echocardiographic variables as predictors (see Supplementary material online, *Appendix S2*).

A complex initial decision tree was generated. This tree captured detailed patterns and relationships within the data. To optimize the model and reduce the risk of overfitting, the initial tree was streamlined using built-in cross-validation techniques provided by the Rpart package.

Streamlining involves evaluating a sequence of subtrees obtained by progressively pruning the initial tree. Each subtree is associated with a cost-complexity measure, which balances the tree’s complexity with its error rate. The cross-validation steps allowed for the evaluation of the subtrees’ performance by splitting the data into training and validation sets multiple times. This process ensures that the model generalizes well to unseen data.

### Statistical analysis

Categorical variables are described as frequencies (percentages) and continuous variables as means ± standard deviation and medians (25th and 75th percentiles). Comparison of patient characteristics across echocardiographic phenotypes was carried out using the Kruskal–Wallis test for continuous variables and χ² or Fisher’s exact test for categorical variables. In multivariable analysis, the most relevant echocardiographic parameters of both ventricles with <10% missing data were incorporated. Binary logistic regression was performed, and results expressed as odds ratio (OR) with 95% confidence interval (CI). All variables included in the multivariable analysis had a Pearson correlation <0.8 between one another.

In addition, a clinical model was created to test the added value of our machine-learning approach on top of existing tools, inspired by the RAISE score and resumed in *Figure 1*, a score ≥ 2 was considered to indicate ATTR-CM.^2^ To assess the consistency of RV hypertrophy evaluation, a subgroup of 30 patients was randomly selected and analysed twice for intra-observer analysis. To examine inter-observer variability, an experienced cardiologist independently measured RVFWT.

C-statistic, integrated discrimination improvement, and continuous net reclassification improvement (NRI) values were calculated to assess the additional diagnostic value of our algorithm on top of the clinical model. A *P* value < 0.05 was considered statistically significant.

## Results

### Baseline characteristics

Baseline clinical and biological characteristics of the 111 patients with and the 149 patients without ATTR-CM are listed in *Table 1*. Patients with ATTR-CM were significantly older (81 ± 9 vs. 74 ± 15 y.o., *P* < 0.001), were predominantly male (76 vs. 48%, *P* < 0.001) and more frequently had a history of carpal tunnel (75 vs. 51%, *P* = 0.018) compared with controls. Comorbidities including elevated blood pressure (61 vs. 72%, *P* = 0.067) or diabetes (17 vs. 29%, *P* = 0.026) were less frequent in patients with ATTR-CM than in the control group. Renal function and signs of heart failure were not significantly different across groups. Controls are mainly hypertensive cardiomyopathy (116, 78%), aortic stenosis cardiopathy (22, 15%), dilated cardiomyopathy (4, 2.7%), ischaemic cardiomyopathy (4, 2.7%), TTR amyloidosis without cardiomyopathy (4, 2.7%) hypertrophic cardiomyopathy (2, 1.3%), hydroxychloroquine cardiomyopathy (1, 0.7%), and unknown cardiomyopathy (1, 0.7%).

**Table 1:** Baseline characteristics of the initial population according to bone scintigraphy-based ATTR-CM diagnosis

Variables	Negative BS (*n* = 149)	ATTR-CM (*n* = 111)	*P*-value
**Clinical characteristics**			
Age, years	74 ± 15	81 ± 9	<0.001
Sex, male, %	71 (48)	84 (76)	<0.001
BMI, kg/m²	26.4 ± 5.8	25.1 ± 3.7	0.027
Arterial hypertension, %	107 (72)	67 (61)	0.07
Diabetes, %	43/148 (29)	18/106 (17)	0.026
CAD, %	38/149 (26)	28/107 (26)	0.9
TTR gene mutation^a^, %	2/13 (15)	6/68 (9)	0.61
V30M	1/13 (8)	2/68 (3)	
V122I	1/13 (8)	2/68 (3)	
P64I		1/68 (1)	
A56P		1/68 (1)	
**Symptoms**			
Peripheral neuropathy, %	11/113 (9.7)	11/69 (16)	0.21
CTS, %	19/37 (51)	47/63 (75)	0.018
Dyspnoea			0.67
NYHA I	31 (21)	26/108 (24)	
NYHA II	70 (47)	52 (49)	
NYHA III	40 (27)	22 (21)	
NYHA IV	8 (5)	7 (6)	
Syncope/Lipothymia, %	11/130 (8.5)	11/84 (13)	0.28
Signs of heart failure, %	27 (18)	26/110 (24)	0.28
**Electrocardiogram**			
Atrial fibrillation, %	60 (40)	77 (69)	<0.001
Paroxystic	29/60 (48)	19/77 (25)	0.69
Persistent	9 (15)	25 (32)	<0.001
Permanent	21 (35)	31 (40)	<0.01
R/LBBB, %	37/145 (26)	30/108 (28)	0.69
LVH (Sokolow), %	27 (19)	7 (7)	<0.01
Pacemaker, %	30/149 (20)	35/109 (32)	0.029
ICD, %	3/149 (2)	8/109 (7)	0.058
**Laboratory findings**			
Creatinine, mg/L	13 ± 11	13 ± 8	0.033
eGFR, mL/min/1.73m²	61 ± 26	59 ± 21	0.4
BNP, pg/mL	551 ± 674	628 ± 678	0.028
NT-proBNP, pg/mL	8974 ± 11 700	5390 ± 11 661	0.70
Natriuretic peptides Z-score	−0.10 (1.14)	0.13 (0.77)	0.082
BNP > 400 or NT-proBNP > cut-off^b^	55/114 (48)	59/91 (65)	0.018
Ic-troponin > 0.05 or HS-troponin > 65 ng/L, %	27/115 (23)	51/80 (64)	< 0.001
Haemoglobin, g/dL	12.7 ± 2.1	13.2 ± 1.7	0.13
Haematocrit, %	39.2 ± 5.9	40.9 ± 4.8	0.2

ATTR-CM, transthyretin amyloid cardiomyopathy; BMI, body mass index, BNP, brain natriuretic peptide, BP, blood pressure, BS, bone scintigraphy, CAD, coronary artery disease, eGFR, estimated glomerular filtration rate, CTS, carpal tunnel syndrome, HR, heart rate, ICD, implantable cardiac defibrillator, LBBB, left bundle branch block, LVH, left ventricular hypertrophy, NYHA, New York Heart Association; RBBB, right bundle branch block, TTE, transthoracic echocardiography.

^a^Number of patients with a pathogenic TTR variant/number of patients tested (positivity rate).

^b^NT-proBNP cut-off: <50 years (450 pg/mL), between 50 and 75 years (900 pg/mL), and >75 years (1800 pg/mL).

### Echocardiographic characteristics

Patients with ATTR-CM had higher LV mass (LV mass index 168 ± 48 vs. 129 ± 43 g/m², *P* < 0.001), concentric pattern (RWT 0.8 ± 0.3 vs. 0.6 ± 0.2, *P* < 0.001), and RV free wall thickness (RVFWT 7.2 ± 1.9 vs. 5.0 ± 1.4 mm, *P* < 0.001) than patients without ATTR-CM (*Table 2*). Patients with and without ATTR-CM did not display significantly different diastolic function patterns or pulmonary pressure levels. Left ventricular ejection fraction (LVEF) and global longitudinal strain (GLS) were lower in ATTR-CM patients compared with controls (47 ± 11 vs. 54 ± 12%, *P* < 0.001 and 11.0 ± 3.7 vs. 14.2 ± 4.5%, *P* < 0.001, respectively). Derived myocardial deformation parameters were also significantly different between the two groups, notably RALS (1.5 ± 1.2 vs. 0.9 ± 0.4, *P* < 0.001), SAB (6.5 ± 5.0 vs. 3.7 ± 5.0, *P* < 0.001) and EFSR (4.5 ± 1.3 vs. 4.0 ± 1.1, *P* < 0.001). When using recommended thresholds, diagnostic accuracy was 0.74 [0.67–0.80] for RALS, 0.71 [0.64–0.77] for SAB and 0.58 [0.51–0.65] for EFSR. In multivariate binary logistic regression, RALS [OR 1.2 (1.1–1.3) for each 0.1 ratio increment, *P* < 0.001], right ventricular wall thickness [OR 2.2 (1.7–3.0) for each mm, *P* < 0.001] and LV mass index [OR 1.4 (1.1–2.0) for each 30 g/m², *P* = 0.02] remained independently associated with a higher risk of ATTR-CM (*Table 3*).

**Table 2:** Echocardiographic parameters of the initial population according to bone scintigraphy-based ATTR-CM diagnosis

Variables	Negative BS (*n* = 149)	ATTR-CM (*n* = 111)	*P*-value
**Structural parameters**			
IVS at end-diastole (mm)	14.2 ± 3.6	17.6 ± 3.7	< 0.001
PWT at end-diastole (mm)	11.9 ± 3.1	15.6 ± 3.9	< 0.001
LV end-diastolic dimension (mm)	45.3 ± 8.6	43.0 ± 7.3	0.035
LV end-systolic dimension (mm)	32.6 ± 9.2	31.8 ± 7.9	0.7
LV mass index (g/m²)	129 ± 43	168 ± 48	< 0.001
LV relative wall thickness (%)	0.61 ± 0.22	0.81 ± 0.26	< 0.001
LVEDV index (mL/m²)	46 ± 20	44 ± 15	>0.9
LVESV index (mL/m²)	22 ± 14	24 ± 12	0.047
**Function parameters**			
LVEF (%)	54 ± 12	47 ± 11	< 0.001
LV fractional shortening (%)	29 ± 11	26 ± 10	0.012
MCF (%)	64 ± 29	40 ± 18	< 0.001
Stroke volume index (mL/m²)	40 ± 13	33 ± 10	< 0.001
Cardiac index (L/min/m²)	2.92 ± 0.82	2.4 ± 0.66	< 0.001
Mean LV GLS (%)	14.1 ± 4.5	11.0 ± 3.7	< 0.001
Mean LV apical LS (%)	18 ± 7	17 ± 6	0.3
Mean LV mid LS (%)	13.1 ± 4.5	9.9 ± 3.8	< 0.001
Mean LV basal LS (%)	10.1 ± 4.8	4.7 ± 3.6	< 0.001
RALS	0.9 ± 0.4	1.5 ± 1.2	< 0.001
SAB	3.7 ± 5.0	6.5 ± 5.0	< 0.001
EFSR	3.98 ± 1.12	4.51 ± 1.32	< 0.001
**Diastolic parameters**			
MV E/A ratio	1.14 ± 0.71	1.81 ± 1.04	< 0.001
MV deceleration time (ms)	207 ± 89	177 ± 60	0.006
MV E’ lateral (cm/s)	8.36 ± 3.04	6.99 ± 2.21	< 0.001
MV E’ septal (cm/s)	6.3 ± 2.3	5.13 ± 1.77	< 0.001
MV E/E’ lateral	10.9 ± 5.1	12.9 ± 6.4	0.009
LA area (cm²)	23 ± 8	26 ± 6	< 0.001
LAV index (mL/m²)	48 ± 22	52 ± 17	0.031
**Valvular parameters**			
Mean transaortic valvular gradient (mmHg)	14 ± 15	7 ± 9	< 0.001
Severe AS, %	21 (14)	5 (4.5)	0.011
Significant MR, %	2 (1.3)	4 (3.6)	0.4
AVR^a^, %	7 (4.6)	8 (7.1)	0.4
**Right ventricle**			
Tricuspid valve S wave (mm)	12.0 ± 3.3	10.6 ± 4.1	< 0.001
TAPSE (mm)	20.3 ± 5.6	16.2 ± 5.6	< 0.001
RVFWT (mm)	4.99 ± 1.41	7.23 ± 1.87	< 0.001
RA area (cm²)	17 ± 7	21 ± 7	< 0.001
Peak TR velocity (m/s)	2.8 ± 0.5	2.8 ± 0.5	0.4
Peak RV-RA gradient (mmHg)	33 ± 12	32 ± 11	0.5
Pericardial effusion (%)	10/148 (6.8)	10/111 (9.1)	0.5

AVR, aortic valve replacement, EFSR, ejection fraction strain ratio, GLS, global longitudinal strain, IVS, interventricular septum, LA, left atrium; LAV, left atrial volume, LV, left ventricle, LVEDV, LV end-diastolic volume, LVEDS, LV end-systolic volume, LVEF, left ventricular ejection fraction, LVOT VTI, left ventricular outflow tract velocity time integral, MCF, Myocardial Contraction Fraction, MR, mitral regurgitation, MV, mitral valve, PWT, posterior wall thickness, RA, right atrium, RALS, relative apical longitudinal sparing, RVFWT, right ventricular wall thickness, RV, right ventricle, SAB, septal apical to base ratio, TAPSE, tricuspid annular plane systolic excursion, TR: tricuspid regurgitation.

^a^AVR (aortic valve replacement) including TAVR (trans aortic valve replacement), biological and mechanical prosthetic.

**Table 3:** Multivariate binary logistic regression assessing performance of echocardiographic parameters for the diagnosis of ATTR-CM

			Multivariable model with LVEF and GLS		Multivariable model with RALS and EFSR	
Variables	Univariable OR (95% CI)	*P*-value	Adjusted OR (95% CI)	*P*-value	Adjusted OR (95% CI)	*P*-value
**LV RWT**. For each 0.1 increment.	1.4 (1.2–1.6)	<0.001	1.2 (1.0–1.4)	0.07	1.1 (0.9–1.3)	0.45
**LV mass index** (g/m²). For each 30 g/m² increment.	1.8 (1.4–2.1)	<0.001	1.4 (1.0–2.0)	0.02	1.5 (1.1–2.1)	0.008
**RVFWT** (mm). For each 1 mm increment.	2.5 (2.0–3.2)	<0.001	2.2 (1.7–3.0)	< 0.001	2.2 (1.7–3.0)	< 0.001
**LA area** (cm²). For each 5cm² increment.	1.2 (1.0–1.5)	0.02	1.0 (0.7–1.4)	0.90	0.9 (0.6–1.3)	0.64
**RA area** (cm²). For each 5cm² increment.	1.7 (1.3–2.0)	<0.001	1.2 (0.8–1.7)	0.31	1.2 (0.9–1.7)	0.24
**TAPSE** (mm). For each 5 mm increment.	0.5 (0.4–0.7)	<0.001	0.7 (0.5–1.1)	0.12	0.7 (0.5–1.0)	0.08
**LVEF** (%). For each 5% increment.	0.8 (0.7–0.9)	<0.001	1.0 (0.8–1.3)	0.84		
**GLS** (%). For each 5% increment.	0.4 (0.3–0.6)	<0.001	0.6 (0.3–1.3)	0.20		
**RALS**. For each 0.1 increment.	1.2 (1.1–1.3)	<0.001			1.2 (1.1–1.3)	< 0.001
**EFSR**. For each 1 increment.	1.4 (1.2–1.8)	0.001			1.2 (0.8–1.7)	0.47

CI, confidence interval; EFSR, ejection fraction strain ratio; GLS, global longitudinal strain; LA, left atria; LV, left ventricle; LVEF, left ventricular ejection fraction; OR, odds ratio, RA, right atria; RALS, relative apical longitudinal sparing; RVFWT, right ventricular free wall thickness; RWT, relative wall thickness; TAPSE, tricuspid annular plane systolic excursion.

### Decision tree for identifying ATTR-CM: GRAAL algorithm

**Figure 2:**
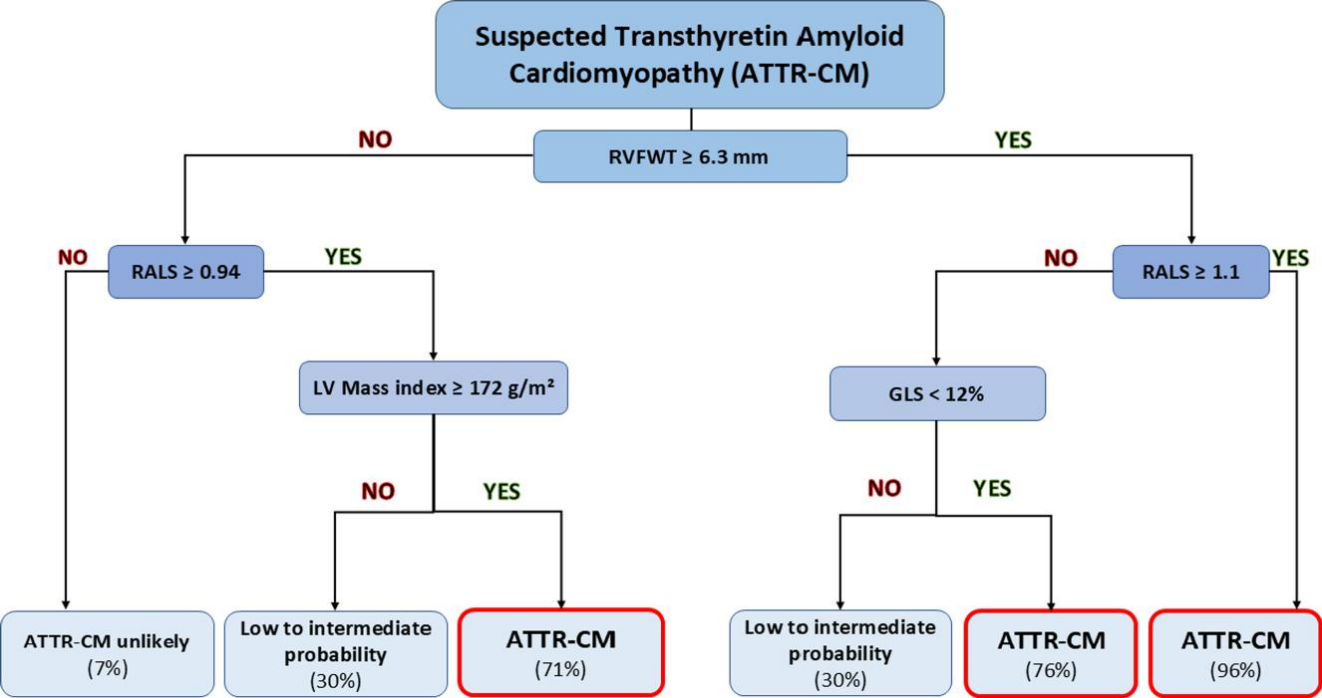
GRAAL algorithm for ATTR-CM diagnosis. % expressed ATTR-CM probability. ATTR-CM: transthyretin amyloid cardiomyopathy, GLS: global longitudinal strain, LV: left ventricle, RVFWT: right ventricular free wall thickness, RALS: relative apical longitudinal sparing.

The supervised decision tree–based machine-learning algorithm identified RV free wall thickness (RVFWT ≥ 6.3 mm), relative apical longitudinal sparing (RALS ≥ 0.94), global longitudinal strain ( < 12%), and left ventricular mass index (≥172 g/m²) as the four most discriminative echocardiographic parameters for identifying ATTR-CM (*Figure 2*). The diagnostic performance of this simple algorithm was good with an AUC of 0.90 [0.86–0.94], *P* < 0.001 (91% sensitivity, 76% specificity). The name GRAAL for **G**LS, **R**VFWT, **A**pical sp**A**ring, **L**V Mass was attributed to this algorithm. LV mass index was available in 99% (258), GLS and RALS in 98% (256) and RVFWT in 97% (253) of the initial cohort—all 4 variables were available in 96% (249) of patients. The intra-observer intraclass correlation coefficient (ICC) for RVFWT measurement was 0.87 and the inter-observer ICC was 0.72. Patients with RVFWT ≥ 6.3 mm and RALS ≥ 1.1, representing approximately one fifth (21%) of the entire study population, had a 96% probability of being diagnosed with ATTR-CM. In contrast, patients without an increase in RVFWT and without apical sparing (RALS < 0.94), representing more than one third (37%) of the entire population, had a 93% probability of not having ATTR-CM. Patients with low to intermediate probability (30%) account for 17% and 9% of the population, respectively, and still require further evaluation to assess the risk of ATTR-CM.

**Figure 3:**
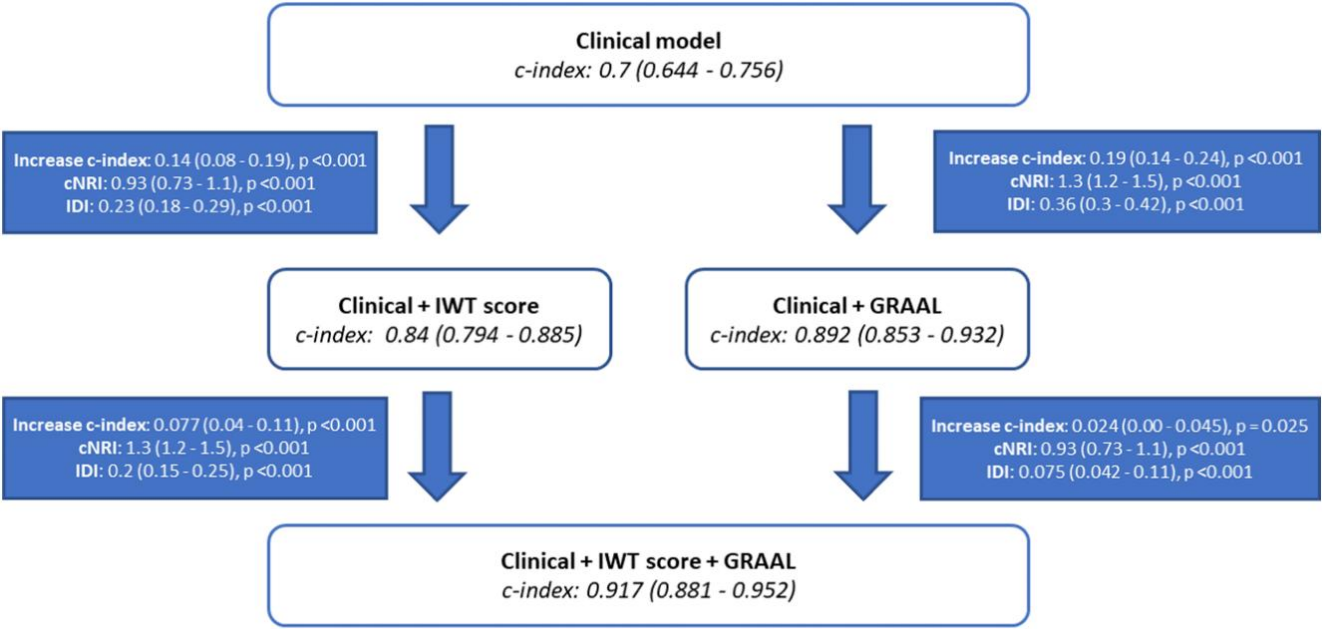
GRAAL additional value on top of clinical and IWT score. GRAAL: GLS, RVFWT, Apical spAring, LV Mass, IDI: integrated discrimination improvement, IWT: increase wall thickness, NRI: net reclassification improvement.

The additional value of the GRAAL algorithm was compared with the IWT score and a clinical model derived from the RAISE score. The clinical model had an AUC for ATTR-CM diagnosis of 0.70 [0.64–0.76], whereas the IWT score (using a threshold of ≥ 8) had an AUC of 0.73 [0.68–0.78] (53% sensitivity, 93% specificity, 86% PPV, and 73% NPV). The GRAAL algorithm significantly improved the diagnostic performance for ATTR-CM on top of the clinical model (increase in C-index of 0.19, *P* < 0.001) as well as on top of the clinical model and IWT score (increase in C-index of 0.08, *P* < 0.001) (*Figure 3*). In addition, the GRAAL algorithm significantly improved the diagnostic performance for ATTR-CM on top of natriuretic peptides [increase in C-index of 0.40 (0.32–0.49), *P* < 0.001].

### Head-to-head comparison between the GRAAL algorithm and the IWT score

**Figure 4:**
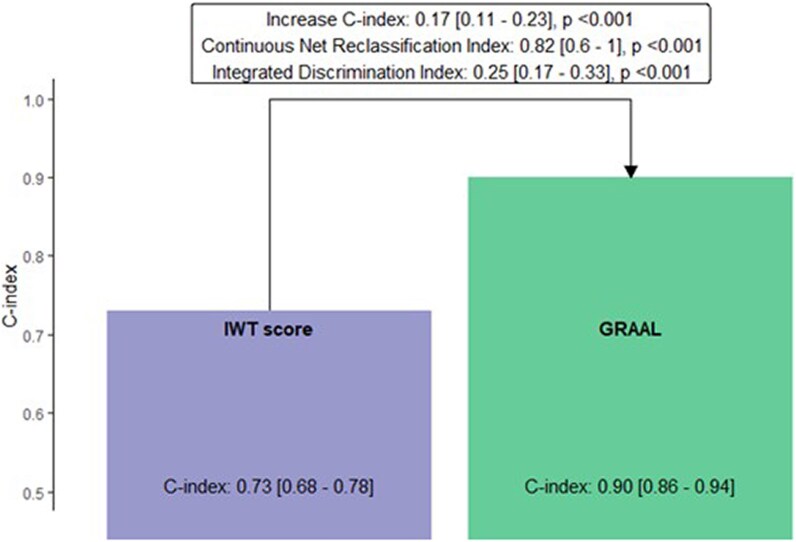
GRAAL performance compared with IWT score. GRAAL: GLS, RVFWT, Apical spAring, LV Mass, IDI: integrated discrimination improvement, IWT: increase wall thickness, NRI: net reclassification improvement.

The added value of the GRAAL algorithm on top of the clinical model was higher [increase in C-index of 0.19 (0.14–0.24)] than that observed with the IWT score [increase in C-index of 0.14 (0.08–0.19)] (*Figure 3*). In addition, the individual C-index of the GRAAL algorithm [0.90 (0.86–0.94)] was significantly higher than the C-index observed for the IWT score [0.73 (0.68–0.78)] (*P* < 0.001) (*Figure 4*).

### External validation cohort

The population of the validation cohort shared similar age (76 ± 13 vs. 77 ± 13 y.o., *P* = 0.5), but had a significant higher male incidence (69 vs. 60%; *P* = 0.01) due to a higher percentage of ATTR-CM diagnoses (57 vs. 43%, *P* < 0.001). Characteristics of the ATTR-CM patients of the validation cohort were similar to those of the initial cohort in terms of age (79 ± 11 vs. 81 ± 9 y.o., *P* = 0.2) and sex ratio (79 vs. 76% male; *P* = 0.5). The echocardiographic characteristics of the ATTR-CM population in the initial and validation cohorts are presented in Supplementary material online, *Table S1*. The diagnostic performance of the GRAAL algorithm in this validation cohort was also within the same range [AUC 0.83 (0.80–0.87), 87% sensitivity, 65% specificity] as that observed in the initial cohort.

## Discussion

In this population of non-selected patients with suspected ATTR-CM, our main findings were the following: (i) the use of apical sparing alone for the detection of ATTR-CM can be misleading; (ii) a simple algorithm using GLS, RVFWT, RALS, and LV mass index (GRAAL algorithm) identify a subgroup of patients with a high probability of ATTR-CM; and (iii) our algorithm improved diagnostic performance on top of recommended echocardiographic scores.

### Overlap of cardiac phenotypes in patients with and without ATTR-CM

At the time of their initial publication, derived myocardial deformation parameters, namely RALS, SAB and EFSR, were presented as revolutionary markers for ATTR-CM diagnosis.^5,6,16^ In our cohort of highly suspected ATTR-CM, these parameters, when taken individually, had a lower diagnostic value. The diagnostic performance of RALS was consistently lower in both our initial and validation cohorts (AUC 0.74 and 0.73), as well as SAB in our initial cohort (AUC 0.70). EFSR was underwhelming in our initial cohort (AUC 0.58) and was consistent with the finding of Boldrini *et al.* in the IWT cohort.^6^ This shortcoming in deformation-derived parameters performance is mainly due to the difference in pre-test probability and the level of structural and functional parameters in the group used as reference. Indeed, an apical sparing pattern has been observed in both chronic kidney disease populations and AS populations with subsequently lower diagnostic performance.^9,10^ This moderate value of apical sparing to adequately identify CA is in line with the recently published results from Cotella *et al.* (72% sensitivity and 66% specificity).^11^

We confirm that patients suspected of having ATTR-CM, whether ultimately diagnosed with ATTR-CM or not, shared many similarities in terms of LV remodelling. These phenotypic overlaps reduce the diagnostic value of echocardiographic parameters, even specific ratios, when taken individually. The failure of isolated echocardiographic parameters to confirm ATTR-CM prompted several authors to propose a multiparametric approach.^2,6^ Expert consensus highlighted an echocardiography score for CA diagnosis (IWT score) which presents a moderate diagnosis performance in our cohort (AUC 0.73).^7^ This score using a 10-point scale with a diagnostic threshold set at ≥8 with different echocardiographic components included RWT, E/e’ ratio, TAPSE, GLS, and SAB. In our population, the IWT score was inconclusive in almost two thirds of the undiagnosed ATTR-CM population, which would consequently have required additional diagnostic tests. This may be related to the predominance of SAB in this score which seems less precise than RALS in our cohort and the fact the score was design for CA diagnosis, regardless of amyloid type. The high rate of false-negatives, consistent with previously reported external validation, may be related to the poor sensitivity (53%) of the IWT score.^17^ This lower diagnostic value is also related to difference of clinical context: the original study evaluates a mixed population (AL and ATTR-CM) while our study included only ATTR-CM.^7^ An agreement table comparing the IWT score and GRAAL classifications (see Supplementary material online, *Table S2*) was performed and a Cohen’s Kappa [0.38 (0.31–0.45)] indicated a fair agreement which highlights the complementary nature of both approaches. However, our results suggest that the IWT score may not be suitable to detect ATTR-CM in an unselected population with a high pre-test probability and phenotypic overlaps.

### Right ventricular thickness as gatekeeper and GLS alteration as additional steps

The present study shows that RVFWT appears as the strongest parameter to predicting ATTR-CM. Each 1 mm increase in RV thickness increased the risk of having an ATTR-CM confirmation by bone scintigraphy by 120% and represented the first step in discriminating patients in our diagnostic algorithm. Moreover, the combination of RVFWT and RALS provided a very high predictive value (>93%) to confirm or rule out ATTR-CM for half of the patients. There is a growing interest in RV remodelling and function in HF and thus its value in ATTR-CM should not be underestimated.^18,19^ Due to its infiltrative process, the involvement of both ventricles is uniform, as opposed to chronic pressure overload which induces LV remodelling while preserving the right ventricle. RV infiltration by TTR deposits is well known and easy to show directly with endomyocardial biopsy or indirectly with the similar relative apical sparing GLS pattern and RV late gadolinium enhancement observed in MRI.^20,21^ While the diagnostic and prognostic value of RV involvement has been reported in AL cardiac amyloidosis,^22,23^ such involvement has only been scarcely assessed in ATTR-CM.^24^ Arvidsson *et al*. reported that 81% of ATTR-CM present increased RVFWT compared with 0–5% of controls and 35% of HCM.^20^

The GRAAL algorithm emphasizes RV remodelling (as a first step) and GLS alteration with apical sparing (as additional steps) that best identify ATTR-CM. The importance of apical sparing to identify ATTR-CM is well-known but it is important to keep in mind that it could be taken by default, especially at the late stage of the disease.^9^ Indeed, the progression of the TTR deposits leads to a global alteration of GLS and a progressive decrease of the specific apical sparing. The GRAAL algorithm’s cut-off threshold for GLS closely mirrors the one reported by Boldrini *et al.* (12% vs. 13%), suggesting the possibility of refining diagnoses in advanced cases where apical sparing can misdiagnose.

### Potential of the GRAAL algorithm in routine clinical practice

The GRAAL algorithm was developed using a supervised decision tree–based machine-learning approach to identify the most informative echocardiographic markers for suspected ATTR-CM. Four key parameters—global longitudinal strain (**G**LS), right ventricular free wall thickness (**R**VFWT), **A**pical sp**A**ring, and **L**eft ventricular mass index—were integrated into a simple, easy-to-remember acronym: GRAAL. This multiparametric, imaging-centred model demonstrated strong diagnostic performance (AUC = 0.90) using evidence-based thresholds derived without prior assumptions.

Compared with the established IWT score, GRAAL significantly improved diagnostic accuracy [C-index gain of 0.17 (0.11–0.23), *P* < 0.001]. In a simulated cohort of 100 patients with a 20% ATTR-CM prevalence, GRAAL identified approximately five additional true positive cases, with improved sensitivity (69% vs. 42%), slightly reduced specificity (89% vs. 93%), and high predictive values (PPV: 62%; NPV: 91%).

While several screening tools have been proposed—particularly in HFpEF cohorts—most rely on hypothesis-driven models without strain imaging, apical sparing, or machine learning, and often lack external validation.^25–27^ In contrast, GRAAL was externally validated in a large multicentre cohort of patients referred for suspected ATTR-CM, where it maintained robust performance (AUC = 0.83). Notably, the score was applicable in over 96% of cases using standard echocardiographic views, supporting its feasibility in routine clinical practice.

Machine-learning–based tools such as GRAAL represent a promising step forward in the precision diagnosis of ATTR-CM.^28,29^ When combined with broader clinical pre-screening strategies (e.g. using administrative data or HFpEF phenotyping), GRAAL could serve as a second-step tool to refine diagnostic suspicion and guide further confirmatory testing.^12^ Although developed in a population with high pre-test probability, GRAAL reflects real-world diagnostic pathways, where patients are referred at various disease stages.

## Limitations

This study has several limitations. First, its cross-sectional design and the selection of patients referred to specialized centres for suspected amyloidosis led to a cohort with a high pre-test probability of ATTR-CM. Similarly, the external validation cohort had a high prevalence of disease, which does not reflect typical screening populations. This enriched case mix may have contributed to an overestimation of diagnostic performance and limits generalizability to unselected populations.

Although GRAAL was intentionally developed using routinely obtainable echocardiographic parameters, reliable assessment of these indices requires adequate acoustic windows and appropriate operator expertise. RVFWT showed only moderate interobserver reproducibility (ICC 0.72), largely due to challenges in consistently delineating the right ventricular free wall; thus, the high feasibility achieved in our cohort (>96%) may not be reproducible in all settings. Accordingly, GRAAL should be applied only when echocardiographic image quality allows accurate and consistent measurements. Future iterations may explore multimodal integration with clinical and advanced imaging data.

RV free wall thickening may also occur in other storage disorders, particularly Anderson–Fabry disease. However, Fabry patients generally present at a younger age, with less pronounced RV hypertrophy, absence of classic apical sparing, and a characteristic inferolateral strain pattern. Biomarkers (α-Gal A, lyso-Gb3) and cardiac MRI (T1 mapping) remain essential when this diagnosis is suspected. Importantly, GRAAL is not intended to differentiate ATTR-CM from other infiltrative or storage cardiomyopathies, but rather to refine echocardiographic detection of ATTR-CM among patients already referred for suspected amyloidosis.

Some Perugini grade 1 patients were classified as controls; since 13 of them did not undergo biopsy, occult cardiac amyloidosis (ATTR or AL) cannot be fully excluded. In the validation cohort, SAB was not available in all patients, precluding complete comparison with the IWT score. Patients with AL cardiac amyloidosis were not included, in line with previous studies focused on ATTR-CM detection.

Only eight patients had hereditary ATTR-CM, reflecting our non-endemic population. In addition, genetic testing was not systematically performed (13/149 controls and 68/111 ATTR-CM patients), limiting our ability to fully distinguish hereditary from wild-type forms. Given that ATTRv may present with distinct phenotypes—including earlier onset, different hypertrophy patterns, or milder RV involvement—the performance of GRAAL in hereditary ATTR-CM cannot be robustly assessed. Finally, the algorithm was specifically developed for ATTR-CM and does not apply to AL amyloidosis. While RVFWT reproducibility is moderate, the threshold-based structure of GRAAL likely mitigates substantial misclassification

## Conclusion

In this large cohort of patients with suspected ATTR-CM, the GRAAL algorithm improved diagnostic accuracy using four key echocardiographic parameters reflecting combined LV and RV remodelling and deformation. Its external validation supports broad applicability as a simple, clinically relevant tool to guide further diagnostic testing.

## Supplementary Material

ztag022_Supplementary_Data

## Data Availability

The data underlying this article will be shared on reasonable request to the corresponding author.
