# Supplementary material for: Machine-learning approach on echocardiography to improve the detection of transthyretin amyloid cardiomyopathy: GRAAL algorithm
Source: Eur Heart J Digit Health. 2026 Mar 25;7(3):ztag022. doi: 10.1093/ehjdh/ztag022 (PMC13012819; doi:10.1093/ehjdh/ztag022)
Supplement: ztag022_Supplementary_Data [file ztag022_supplementary_data.pdf]

SUPPLEMENTARY MATERIAL

Appendix 1. Echocardiographic measurements

Two-dimensional echocardiographic images of the left ventricle (LV) were obtained from the parasternal long axis and short axis views and from the three standard LV apical views (4- ,2- and 3-chambers). Conventional echocardiographic (13) parameters were measured including diastolic interventricular septum, posterior wall, and LV diameters from the parasternal long axis view. Relative wall thickness (RWT) was calculated with the formula: SWT + PWTd / LVEDD, where PWTd is the posterior wall thickness at end-diastole and LVVEDD the LV end-diastolic dimension. LVEF was calculated by the Simpson's biplane method from the apical 4- and 2-chamber views. LA volume was measured at end-systole, using LA surface combined in LV apical 4- and 2-chamber views. Peak velocities of early (E) and late (A) waves of the transmitral flow and E-wave deceleration time were measured from pulse-wave Doppler obtained at the tip of the mitral leaflets. The mean of septal and lateral annular velocities (E') was obtained by tissue Doppler imaging (TDI). The E/E′ lateral ratio was calculated using the peak E-wave velocity and averaged lateral E' values (13). Right ventricular free wall thickness (RVFWT) was measured in the subcostal view at the end of diastole, using either M-mode or two-dimensional (2D) imaging. Alternatively, the left parasternal view was also used for measuring RVFWT. Measurement was performed at the middle segment with > 5mm thickness as the cut-off threshold for its increase (14).

All strain measurements were performed offline with dedicated automated software (Q analysis software, Echo PAC® PC version 110.1.0, GE Healthcare). The software allows generating LV strain curves, corresponding to each myocardial segment. Tracking feasibility in each apical view was rated by visual inspection and was carried out in the 4-chamber view only when other apical views were either missing or of poor quality. The operator, when necessary, could adjust the end-systolic frame, the endocardial trace as well as the width of the

region of interest. By convention, end-diastole was defined as QRS onset. As recommended, strain changes were considered using the absolute value of the number (i.e., an increase in GLS signifying that the number is becoming increasingly negative).

Appendix 2. The 22 echocardiographic variables used for the GRAAL algorithm.

The 22 echocardiographic variables used for constructing the GRAAL algorithm were: septal wall thickness, posterior wall thickness, relative wall thickness, LV mass index, LVEF, cardiac index, LVEDV, stroke volume index, LA area, LAV index, E wave, E/A, E/E’ lateral, E lateral, peak TR velocity, TR max gradient, RA area, TAPSE, RVFWT, GLS, RALS, and EFSR.

Supplemental Table 1. Comparison of baseline and echocardiographic characteristics between the initial and validation cohorts of the ATTR-CM population

Variables	Initial cohort (n = 112)	Validation cohort (n = 257)	p-value
	**Mean ±SD / n(%)**	**Mean ±SD / n(%)**	
**Clinical characteristics**			
Age, years	81±9	79 ±11	0.2
Sex, Male (%)	85 (76 %)	203 (79 %)	0.5
**Echocardiographic Parameters**			
**Structural parameters**			
IVS at end diastole (mm)	17.6 ±3.7	16.5 ± 3.6	**0.012**
PWT at end diastole (mm)	15.6 ±4.0	14.8 ±3.2	0.14
LV end-diastolic dimension (mm)	43 ±7.2	44 ± 6.8	0.3
LV Mass index (g/m²)	167 ± 48	165 ± 66	0.091
LV Relative wall thickness (%)	0.80 ± 0.26	0.73 ± 0.21	**0.016**
**Function parameters**			
LVEF (%)	47 ± 11	53 ± 13	< **0.001**

Variables	Initial cohort (n = 112)	Validation cohort (n = 257)	p-value
	**Mean ±SD / n(%)**	**Mean ±SD / n(%)**	
Stroke volume index (mL/m²)	33 ± 10	35 ± 11	0.2
Cardiac index (L/min/m²)	2.4 ± 0.7	2.6 ± 0.8	**0.043**
Mean LV GLS (%)	11 ± 3.7	11.4 ± 3.7	0.2
RALS	1.5 ± 1.16	1.3 ± 0.97	0.4
EFSR	4.5 ± 1.3	5.2 ± 3.5	**0.002**
**Diastolic parameters**			
MV E/A ratio	1.8 ± 1.0	1.6 ± 1.2	**0.031**
MV E' lateral (cm/s)	7.0 ± 2.2	6.6 ± 2.0	0.13
MV E/E' lateral	13 ± 6	17 ± 8	< **0.001**
LA area (cm²)	26 ± 6	25 ± 5	0.3
LAV index (mL/m²)	52 ± 17	54 ± 19	0.5
**Right ventricle**			
TAPSE (mm)	16.2 ± 5.6	17.3 ± 5.1	0.069
RVFWT (mm)	7.2 ± 1.9	7.2 ± 1.6	0.5
RA area (cm²)	21 ± 7	22 ± 7	0.4

Variables	Initial cohort (n = 112)	Validation cohort (n = 257)	p-value
	Mean ±SD / n(%)	Mean ±SD / n(%)	
Peak TR velocity (m/s)	2.8 ± 0.5	2.7 ± 0.5	0.10
Peak RV-RA gradient (mmHg)	32 ± 11	34 ± 13	0.3

EFSR: ejection fraction strain ratio, GLS: global longitudinal strain, IVS: interventricular septum, LA: left atrium, LAV: left atrial volume, LV: left ventricle, LVEF: left ventricular ejection fraction, MV: mitral valve, PWT: posterior wall thickness, RA: right atria, RALS: relative apical longitudinal sparing, RVFWT: right ventricular wall thickness, RV: right ventricle, TAPSE: tricuspid annular plane systolic excursion, TR: tricuspid regurgitation.

Supplementary Table 2: Agreement table comparing the IWT score and GRAAL classifications for the diagnosis of ATTR-CM

	GRAAL Positive	GRAAL Negative	Total
**IWT score Positive**	134	44	178
**IWT score Negative**	157	379	536
**Total**	291	423	714

GRAAL: GLS, RVFWT, Apical spAring, LV Mass; IWT: increase wall thickness.

Supplementary Table 3: Initial cohort characteristics during the earlier (pre-median) versus later (post-median) eras

Characteristic	post-median N = 130[1]	pre-median N = 130[1]	p-value[2]
**ATTR-CM**	55 (42%)	56 (43%)	>0.9
**Sex, male, %**	78 (60%)	77 (59%)	0.9
**Age, years**	78 ± 12	75 ± 14	0.024
**CTS, %**	37 (62%)	29 (73%)	0.3
**Arterial hypertension, %**	91 (72%)	83 (64%)	0.2
**Diabetes, %**	30 (24%)	31 (24%)	0.9
**BMI, kg/m²**	26.4 ± 5.4	25.4 ± 4.6	0.2
**R/LBBB, %**	33 (25%)	34 (26%)	0.9
**IVS at end-diastole (mm)**	1.54 ± 0.38	1.60 ± 0.42	0.2
**RWT**	0.63 ± 0.27	0.66 ± 0.29	0.3
**LV Mass index (g/m²)**	144 ± 49	148 ± 49	0.5

**Characteristic**	**post-median** N = 130[1]	**pre-median** N = 130[1]	**p-value**[2]
**LVEF (%)**	52 ± 12	50 ± 12	0.2
**LVEDV (mL)**	81 ± 36	84 ± 32	0.4
**Tricuspid valve S wave (mm)**	6.46 ± 1.99	6.46 ± 2.06	0.9
**Cardiac index (L/min/m²)**	2.73 ± 0.79	2.66 ± 0.81	0.5
**LAV index (mL/m²)**	49 ± 21	50 ± 19	0.4
**MV E/A ratio**	1.29 ± 0.76	1.43 ± 1.01	0.8
**MV E/E' mean**	12.5 ± 5.2	14.1 ± 5.9	0.073
**MV E' lateral (cm/s)**	8.12 ± 2.94	7.39 ± 2.58	0.071
**Peak TR velocity (m/s)**	2.80 ± 0.55	2.79 ± 0.46	>0.9
**RVFWT (mm)**	6.01 ± 2.13	5.86 ± 1.79	0.5
**Mean LV GLS (%)**	13.3 ± 4.3	12.3 ± 4.5	0.052
**RALS**	1.03 ± 0.52	1.26 ± 1.12	0.4

Characteristic	post-median N = 130[1]	pre-median N = 130[1]	p-value[2]
**EFSR**	4.20 ± 1.21	4.23 ± 1.26	0.4
**Mean RV GLS**	18.1 ± 6.2	18.1 ± 6.2	0.8

[1] n (%); Mean ± SD

[2] Pearson's Chi-squared test; Wilcoxon rank sum test

Pré-median sensitivity of 0.91, specificity of 0.71 and negative predictive value of 0.85 and a post-median sensitivity of 0.92, specificity of 0.8 and negative predictive value of 0.88.

ATTR-CM: transthyretin amyloid cardiomyopathy, BMI: body mass index, CTS: carpal tunnel syndrome, EFSR: ejection fraction strain ratio, GLS: global longitudinal strain, IVS: interventricular septum, LBBB: left bundle branch block, LAV: left atrial volume, LV: left ventricle, LVEF: left ventricle ejection fraction, LVEDV: LV end-diastolic volume, MV: mitral valve, RALS: relative apical longitudinal sparing, RBBB: right bundle branch block, RVFWT: right ventricular wall thickness, RWT: relative wall thickness, RV: right ventricle, TR: tricuspid regurgitation.

Supplementary Table 5: Comparison of baseline characteristics between the initial and validation cohorts

**Characteristic**	**initial**<br>N = 260[1]	**replication**<br>N = 454[1]	**p-value**[2]
**ATTR-CM**	111 / 260 (43)	257 / 454 (57)	<0.001
**Age, years**	77 ± 13	76 ± 13	0.5
**Sex, male, %**	155 / 260 (60)	313 / 454 (69)	0.012
**Signs of heart failure, %**	53 / 260 (20)	135 / 454 (30)	0.006
**Arterial hypertension, %**	174 / 260 (67)	137 / 454 (30)	<0.001
**Diabetes, %**	61 / 260 (23)	25 / 454 (5.5)	<0.001
**CAD, %**	66 / 260 (25)	52 / 454 (11)	<0.001
**NYHA**			<0.001
1	57 / 256 (22)	15 / 210 (7.1)	
2	122 / 256 (48)	107 / 210 (51)	
3	62 / 256 (24)	52 / 210 (25)	

Characteristic	initial<br>N = 260[1]	replication<br>N = 454[1]	p-value[2]
4	15 / 256 (5.9)	3 / 210 (1.4)	
**Syncope / Lipothymia, %**	22 / 260 (8.5)	7 / 454 (1.5)	<0.001
**Peripheral neuropathy, %**	22 / 260 (8.5)	28 / 454 (6.2)	0.2
**CTS, %**	66 / 260 (25)	51 / 454 (11)	<0.001
**BMI, kg/m²**	25.9 ± 5.0	25.3 ± 4.3	0.5
**RBBB**	34 / 260 (13)	29 / 454 (6.4)	<0.001
**LBBBB**	33 / 260 (13)	33 / 454 (7.3)	<0.001
**Atrial fibrillation**	137 / 260 (53)	103 / 454 (23)	<0.001
**Pacemaker**	65 / 260 (25)	40 / 454 (8.8)	<0.001
**Creatinine, mg/L**	13 ± 10	12 ± 6	0.6
**Hemoglobin, g/dL**	12.88 ± 1.98	13.51 ± 1.49	0.001
**Hematocrit, %**	39.9 ± 5.5	42.0 ± 4.7	0.2
**IVS at end-diastole (mm)**	1.57 ± 0.40	1.52 ± 0.40	0.3

Characteristic	initial<br>N = 260[1]	replication<br>N = 454[1]	p-value[2]
**RWT**	0.64 ± 0.28	0.62 ± 0.23	0.9
**LV Mass index (g/m²)**	146 ± 49	147 ± 61	0.5
**LVEF (%)**	51 ± 12	54 ± 13	<0.001
**LVEDV (mL)**	83 ± 34	92 ± 32	0.001
**Tricuspid valve S wave (mm)**	6.46 ± 2.02	6.22 ± 1.62	0.6
**Cardiac index (L/min/m²)**	2.70 ± 0.80	2.74 ± 0.87	0.6
**LAV index (mL/m²)**	50 ± 20	51 ± 20	0.5
**MV E/A ratio**	1.36 ± 0.89	1.35 ± 1.08	0.3
**MV E/E' mean**	13 ± 6	17 ± 8	<0.001
**MV E' lateral (cm/s)**	7.77 ± 2.79	6.98 ± 2.43	0.005
**Peak TR velocity (m/s)**	2.80 ± 0.51	2.66 ± 0.51	0.001
**RVFWT (mm)**	5.94 ± 1.96	6.37 ± 1.82	<0.001
**Mean LV GLS (%)**	12.8 ± 4.4	12.8 ± 4.5	>0.9

Characteristic	initial<br>N = 260[1]	replication<br>N = 454[1]	p-value[2]
**RALS**	1.15 ± 0.88	1.11 ± 0.81	0.8
**EFSR**	4.21 ± 1.24	4.71 ± 2.83	0.001
**Mean RV GLS**	18 ± 6	18 ± 7	0.7

[1] n / N (%); Mean ± SD. [2] Pearson's Chi-squared test; Wilcoxon rank sum test; Fisher's exact test

ATTR-CM: transthyretin amyloid cardiomyopathy, BMI: body mass index, CAD: coronary artery disease, CTS: carpal tunnel syndrome, EFSR: ejection fraction strain ratio, GLS: global longitudinal strain, IVS: interventricular septum, LBBB: left bundle branch block, LAV: left atrial volume, LV: left ventricle, LVEF: left ventricle ejection fraction, LVEDV: LV end-diastolic volume, MV: mitral valve, NYHA: New York Heart Association, RALS: relative apical longitudinal sparing, RBBB: right bundle branch block, RVFWT: right ventricular wall thickness, RWT: relative wall thickness, RV: right ventricle, TR: tricuspid regurgitation.
